# Improved mediolateral load distribution without adverse laxity pattern in robot-assisted knee arthroplasty compared to a standard manual measured resection technique

**DOI:** 10.1007/s00167-019-05631-y

**Published:** 2019-07-27

**Authors:** William Manning, Milton Ghosh, Ian Wilson, Geoff Hide, Lee Longstaff, David Deehan

**Affiliations:** 1 Newcastle Surgical Training Centre Research Unit Freeman Hospital, High Heaton, Newcastle upon Tyne, NE7 7DN UK; 2 Institute of Genetic Medicine, Newcastle University International Centre for Life, Central Parkway, Newcastle upon Tyne, NE1 3BZ UK; 3 University Hospital of North Durham, Durham, DH1 5TW England, UK; 4 Freeman Hospital, High Heaton, Newcastle upon Tyne, NE7 7DN UK

**Keywords:** Robotic, Load, Knee, Laxity, Arthroplasty, Cadaveric

## Abstract

**Purpose:**

Robot-assisted total knee arthroplasty (rTKA) remains in its infancy, is expensive but offers the promise of improved kinematic performance through precise bone cuts, with minimal soft tissue disruption, based on pre-resection soft tissue behaviour. This cadaveric study examined load transfer, soft tissue performance and radiographic indices for conventional (sTKA) versus rTKA. The null hypothesis was there would be no difference between the two modes of implantation.

**Methods:**

Whole (ten) cadaveric limbs were randomised to receive either robotic (rTKA, *N* = 5) or conventional measured resection (sTKA, *N* = 5) knee arthroplasty. Laxity patterns were established using validated fixed sensors (Verasense) with manual maximum displacement for six degrees of freedom. Tibiofemoral load and contact points were determined dynamically using remote sensor technology for medial and lateral compartments through a functional arc of motion (0–110 degrees of motion). Final component position was assessed using pre- and post-implantation CT.

**Results:**

No significant intergroup differences for laxity were found (n.s.). The rTKA group exhibited consistently balanced mediolateral load throughout the full arc with significantly reduced overall total load across the joint (for distinct points of measurement, *p* < 0.05). Despite using flexion–extension and mediolateral gap balancing with measured resection, the sTKA group failed to achieve balance in at least three points of the flexion arc. Post-operative CT confirmed satisfactory component alignment with no significant differences for positioning between the two groups.

**Conclusion:**

This work found improved load sharing for rTKA when compared to conventional surgery for same donor knees. Laxity and CT determined final component positioning was not significantly different. The work supports the contention that robot-assisted TKA delivers improved tibiofemoral load sharing in time zero studies under defined conditions but such offers the promise of improved clinical performance and reduced implant wear.

## Introduction

As the burden of arthroplasty is expected to increase, there remains an unacceptably high rate of patient-reported dissatisfaction [3, 11, 26]. The immediate desired effect is pain relief, the medium goal is optimization of function and survivorship is a long-term objective. Intra-operative and very early clinical measurements such as time to discharge or single-plane alignment determination remain nothing more than surrogate markers for patient-relevant outcomes.

Navigated knee arthroplasty did achieve a tightening of the Gaussian distribution for mechanical alignment without demonstrating reliable improvement in functional outcome perhaps explaining the failure of widespread adoption of such expensive technology [7, 25, 40]. Patient-specific instrumentation was hailed as the answer to matching the anatomy to optimized soft tissue characteristics but has not been shown to consistently improve functional outcome [1, 6]. The debate between mechanical and kinematic alignment continues yet axiomatically single-view stance radiography cannot possibly be extrapolated to whole lower-limb mechanical behaviour [43]. Translation of early outcome improvements for partial may not necessarily be translated into surgery for tricompartmental disease with often more complex soft tissue deformity [13, 33, 46]. The final common pathway for alignment, intra-operative, subjective assessment such as patellar tracking is to achieve equivalence of load across the tibiofemoral joint through full arc of motion using defined axes of motion [15, 21, 30, 35, 45]. Such intra-operative assessment is increasingly being adopted through sensor technology. Such measurements have been shown to be more reliable than the use of tensor devices or standard instrumentation [29].

Headlong haphazard adoption of new technology without determining the value of such for state of the art assessment may be construed as commercially advantageous but may not necessarily be in the best interests of the patient. A key indicator of long term performance may be load distribution across the joint and its impact on the soft tissue envelope. Hence, it was considered desirable to examine through a simple comparative study the influence of robot-assisted surgery upon load distribution when compared with a conventional measured resection surgical technique. The null hypothesis was that there would be no difference for load across the tibiofemoral articulation for all ranges of motion for the two forms of knee arthroplasty.

## Materials and methods

Six whole cadaveric specimens (pelvis to feet) were obtained from a tissue bank (Science Care Phoenix Az 85207). Each cadaver provided two knees upon which to perform TKA. All limbs exhibited full passive movement; documentation from Science Care (Phoenix, AZ. USA) confirmed that there was no record of previous surgery, trauma with no evidence of malalignment or gross torsional abnormality. The limbs were randomized to receive a knee implant performed through either conventional, measured resection or robot-assisted means. The median BMI of the donors was 24.1 (range 20–30) 3 female 3 male, 4 white Caucasian, 1 African–American, median age was 76 years, range 61–85. Power analyses from previous studies and again for this work based on preliminary (three separate cadaveric robotic measured procedures) had determined that five matched pairs of limbs would be sufficient to allow for identification of any significant differences with 95% confidence at 80% power. Pre-operative CT scans from hip to ankle joint were performed on all five pairs of limbs in extension to determine coronal and rotational alignment and to exclude peri-articular or limb segment. All knees underwent the MAKO (MAKO Surgical Corp. Stryker Ft. Lauderdale) CT planning protocol (PN 200004) prior to randomization. One femur fractured beyond salvage and another component sustained significant soft tissue disruption during preparation/surgery thereby allowed for comparison of 10 specimens (5 in each group).

### Specimen preparation and experiment set-up

**Fig. 1:**
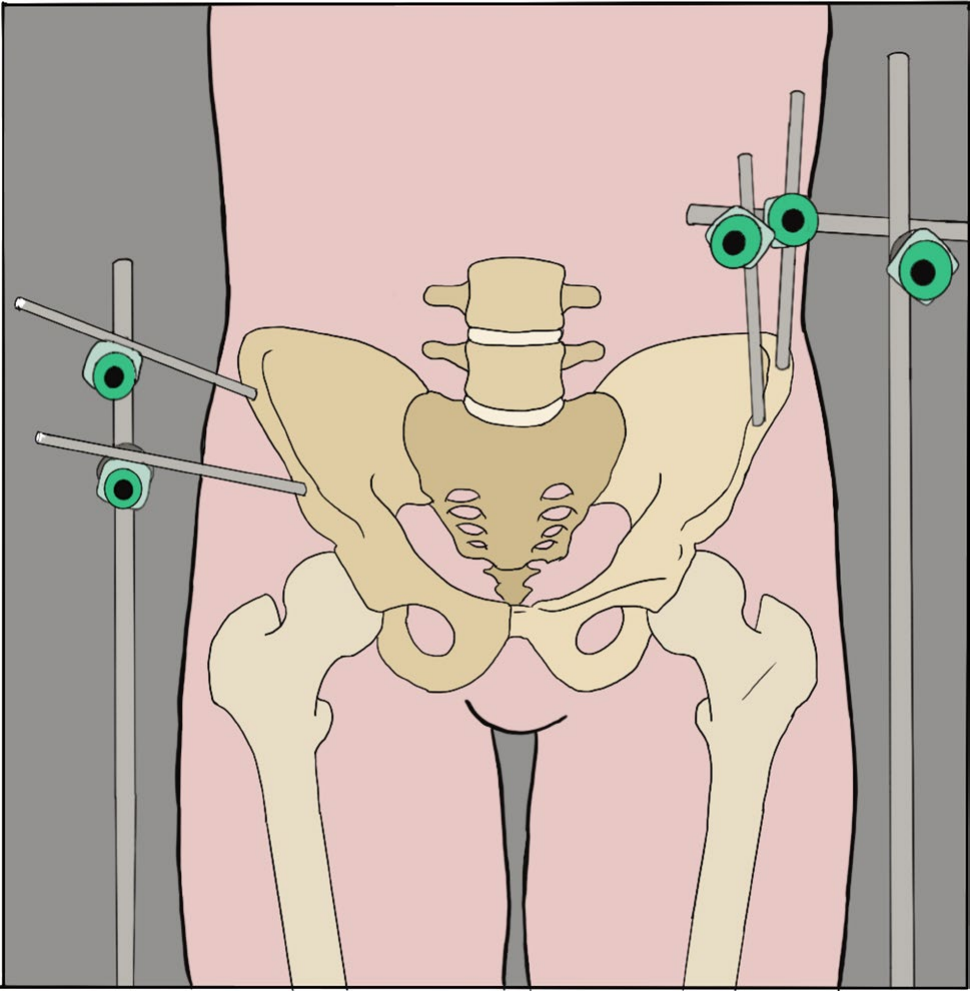
Schematic image of fixation of pelvis

**Fig. 2:**
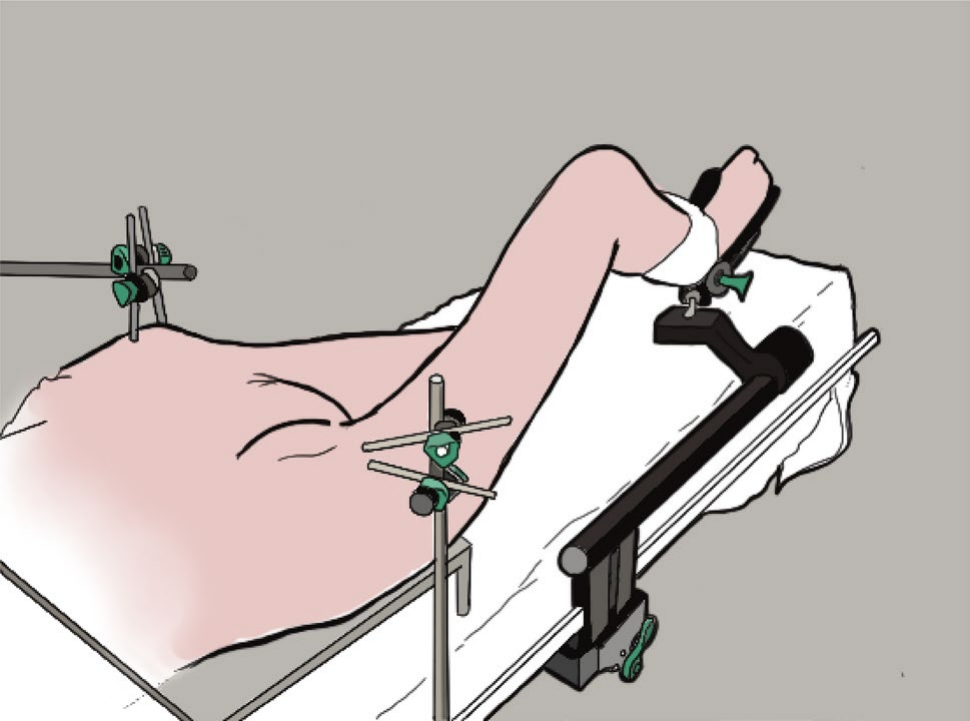
Schematic image of the setup of operated limb with pelvis fixation using external mounted frame and leg holder

A custom rig was built specifically to immobilize the pelvis but allow a full range of hip and knee movements. The rig consisted of two 350 mm-long/11 mm diameter upright stainless steel rods welded to table clamps. When the clamps were secured to the table (on either side of the specimen) the rods acted as vertical “outriggers” to which Hoffman III external fixator (Stryker, Kalamazoo, USA) delta clamps could be attached. A total of four, 5 mm apex half pins (180 mm long) were then driven into the ilium (two into each side) just above the acetabulum to secure the pelvis (Fig. 1). The pelvis was raised on a platform approximately 15 cm off the operating table and the lower limb was secured in a leg positioner (Fig. 2) thereby enabling the index operated knee to be positioned at any point within a full arc of motion. Skin and subcutaneous tissue were then dissected carefully from the proximal half of the thigh and the quadriceps and hamstring muscle groups were separated. Loading of the individual flexor and extensor muscle groups has been previously reported and was performed within previously accepted safe loads in an open chain fashion [12, 22, 28]. Navigation arrays were then secured to the tibia and femur in accordance with the MAKO TKA Surgical Guide. All knees underwent a medial parapatellar approach followed by navigation mapping of the tibial and femoral osteology. Randomization was performed using a sealed envelope technique. Three left and two right knees were randomized to receive robotic (rTKA) implantation and two left and three right knees therefore received a standard measured resection instrumented arthroplasty TKA (sTKA).

### Standard total knee arthroplasty

A single radius cruciate retaining Total Knee Arthoplasty (CR-TKA) (Stryker Triathlon, Michigan USA) was performed via the medial parapatellar approach using a measured resection technique [39]. The transepicondylar axis and central trochlear sulcus were used for femoral component rotational referencing [4]. The tibial rotation for all cases was neutral with respect to a line drawn between the medial aspect of the patellar tendon and the PCL footprint [2, 36]. For all knees the senior authors subjectively deemed the knee balanced upon completion of trialing. Definitive implants were then cemented using Palacos® (Heraeus, Hanau, Germany) bone cement. A trial tibial insert of appropriate thickness was secured before closing the arthrotomy.

### Robotic TKA

Following arthrotomy, anatomical landmarks were referenced using a wand matching the digitised bone model to the CT. Medial and lateral gaps were evaluated in flexion and extension prior using spacer paddles to guide final soft-tissue tension. The CT screen bone model continuously updates in real time, thereby giving feedback on alignment and bone resection so as to achieve rectangular gaps in extension and 90° of flexion. Final positioning of the implant on the bone was determined through rotation of the tibial component. Bone cuts were made with the haptic-controlled arm-mounted oscillating saw guided by navigational arrays. The definitive implant was then cemented into place as per the technique for the sTKA group. No additional soft-tissue releases were required for these robot-assisted procedures.

### Laxity testing

Prior to intervention the native knee was taken through 10 complete flexion/extension cycles to reduce hysteresis and ensure stable, reproducible recordings from the navigation array throughout the arc of flexion (0°–120°). The femur was then secured and fixed using two further half pins attached to the uprights. The position of femoral fixation ensured the tibia (when removed from the leg positioner) could be moved through a full arc of motion (0°–140°). Knees were manually stressed to mimic intraoperative laxity assessment, with the endpoints defined as the maximal displacement achieved [12, 22]. The TKA in extension acted as the datum from which maximal displacements of the tibia in relation to the femur were recorded in six degrees of freedom [14]. For each knee condition, maximal manual varus, valgus, internal and external rotational (IR–ER) displacements were each recorded at five angles of flexion (0°, 30°, 60°, 90° and 120°). Each recording was performed three times.

### Load testing

Following laxity testing and for all knees, the arthrotomy was re-opened and a wireless load sensor Verasense™ (Orthosensor, Dania FL) of the same thickness was activated and replaced the polyethylene trial on the tibial tray [28]. The arthrotomy was then closed and medial and lateral tibiofemoral contact forces were recorded as the loaded knee was taken through a range of passive flexion. The median of each of the three measurements was taken. The use/calibration and limitations of the Verasense™ have previously been reported for both clinical and cadaveric settings [15, 16, 39]. A side-to-side difference of > 66.7 N was classed as not balanced as per Verasense™ guidance [15]. Computer navigation was a Stryker eNdtrac Knee Navigation System, Michigan USA allowing for tracked knee motion to an accuracy of ± 0.5 mm. The average time between two digitizations was approximately 150 ms, which equated to a frame rate of 6.67 Hz m [10]. The Verasense™ tibial sensor recorded tibiofemoral contact force (lbs/force) and contact points (mm accuracy ± 2 mm). This was taken for 0°, 30°, 60°, 90° and 110° of knee flexion.

### Component position

**Fig. 3:**
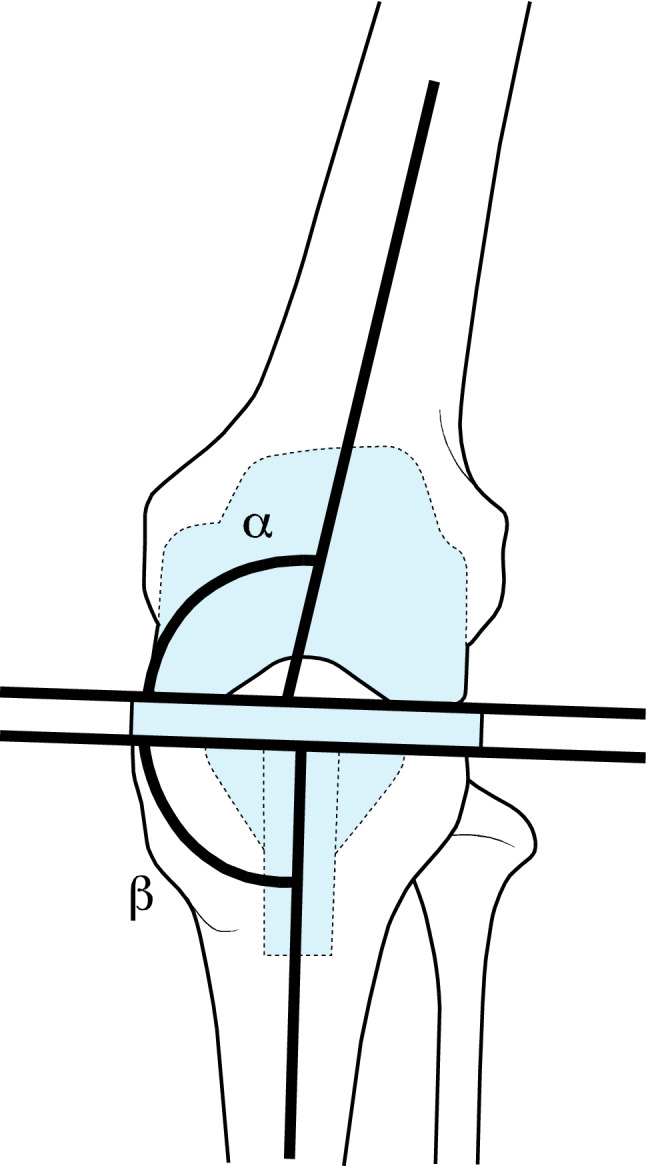
Schematic representation of alpha and beta angles in frontal plane

**Fig. 4:**
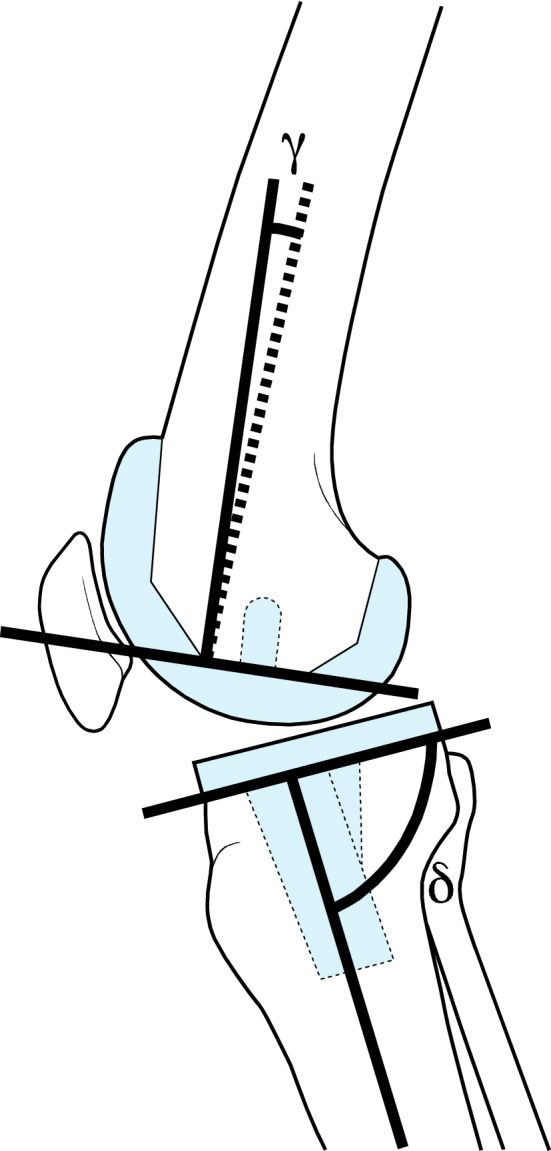
Schematic representation of gamma and delta angles in sagittal plane

Following laxity and load measurements all knees were re-imaged and CT scans were analysed by a blinded senior radiologist to assess component rotation using previously validated protocols [17]. Femoral component rotation was measured as the angle between the posterior condylar line of the femoral component and the trans-epicondylar axis. A positive (+ve) value was given for external rotation. Tibial component rotation was measured by the angle between a line drawn from the centre of the footprint of the PCL and the medial border of the tibial tuberosity and a line perpendicular to the line connecting the posterior fins of the tibial base plate [37]. A positive (+ve) value was given for external rotation. Tibiofemoral component divergence (matched rotation) was calculated using lines perpendicular to the femoral posterior condylar axis and a line perpendicular to the line connecting the posterior fins of the tibial base plate. A positive (+ve) value was given for external rotation of the tibial component relative to the femoral component. Radiographic measurement of divergence for femoral/tibial component alignment in the coronal plane, (*α*, *β* angles) (Fig. 3) and in the sagittal plane (*γ*, *δ* angles) (Fig. 4) were determined from each post implantation CT scan.

### Statistical analysis

The numbers used were based on precedent and a pre-hoc determination was made from previous power analysis in multiple papers on laxity for defined differences in varus valgus and rotational laxity. Load analysis and differences were performed based on preliminary experiments (not included in the final numbers) examining the measured load across the tibiofemoral articulation. Effect sizes determined from previous studies indicated that eight limbs in total (for laxity and pre and post load) were sufficient to identify significant differences in load transfer and laxity with 95% confidence at 80% power. The previous studies examined a total of 8 but in this work each limb acted as a control combining pre and post laxity patterns and the changes from pre to post were examined for the two groups. In effect, each limb acted as its own control. Three replicate measurements were taken for all measurements of angle, load at each point of flexion and the median has been used for simple statistical summaries at each point of measurement through flexion arc. Linear mixed-effect models were used to model the relation between the measured outcomes (laxity angles and load) and knee flexion [34]. All three outcome measurements were used to account for uncertainty at each flexion angle. This approach accounts for the structure of these data, where values taken at each knee are expected to be more correlated than between knees, and measurements taken at different angles within the same knee are correlated. Comparison of knees before arthroplasty was performed for varus, valgus, internal and external rotation using fixed effects for operation type (rTKA versus sTKA) and flexion. Tests of difference between MAKO and manual knees were performed post implantation thereby testing for differences in overall effect and for the change with defined point of flexion angle. Random effects for both intercept and slope (relationship of outcome variable to flexion) were included for varus, valgus, internal and external angle measurements. Quadratic random effects were included for all loading variables to account for the curvilinear relationship between all loading outcome measures and flexion which differed for each knee. General linear mixed model (GLMM) linear models were fitted using maximum likelihood and statistical significance assessed using likelihood ratio tests, using a normal error model. Statistical analyses were done using R [34], the R libraries lme4 [4] and lmerTest [27]. A type 1 error probability was taken as 0.05

## Results

Data are given as a mean with standard error.

### Rotational laxity (Fig. 5)

Using the GLMM, no significant differences were found for rotational laxity or varus–valgus maximal laxity in the pre-operative knees randomly assigned to sTKA and rTKA. No significant difference was found for maximal internal or external rotational laxity between the two completed states (sTKA and rTKA) at any of the defined points of flexion. There was a consistently greater standard deviation for the conventionally implanted knee arthroplasty compared to the robot-assisted arthroplasty.

**Fig. 5:**
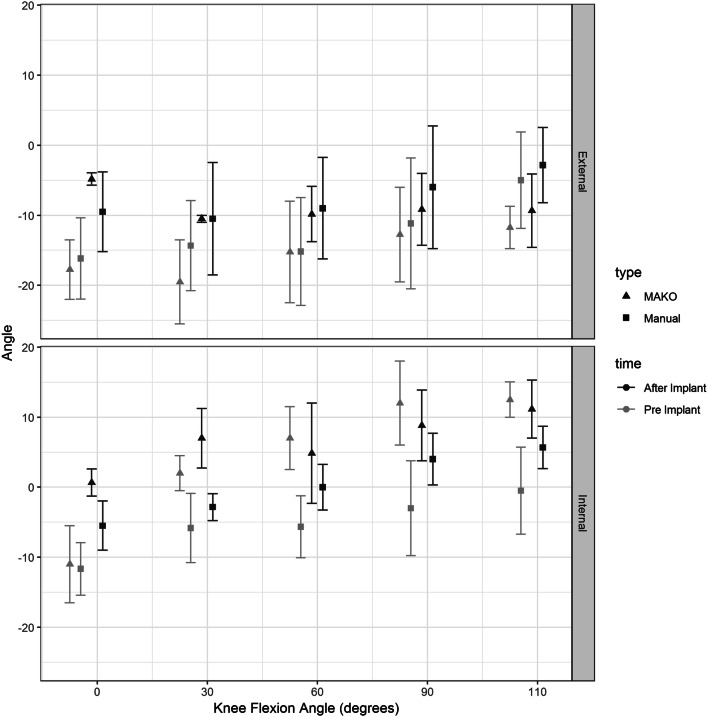
Maximal external and internal rotational displacement in degrees by knee flexion angle for robot-assisted (MAKO) versus conventional (manual) total knee arthroplasty constructs. Black triangles represent MAKO and squares represent manual measurement

### Varus—Valgus maximal laxity (Fig. 6)

There was no significant difference between rTKA or sTKA for maximal varus or valgus laxity for any of the points of knee flexion through the measured arc of motion.

**Fig. 6:**
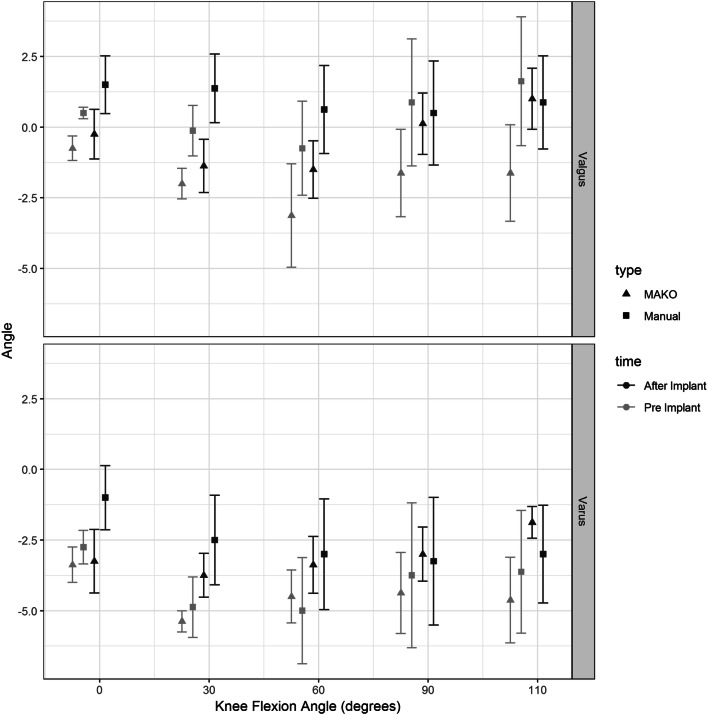
Maximal varus and valgus displacement in degrees by knee flexion angle for robot-assisted (MAKO) versus conventional (manual) total knee arthroplasty constructs. Black triangles represent MAKO and squares represent manual measurement

### Load (Fig. 7)

There were no significant differences between rTKA and sTKA for total loads. Near full extension there was greater load on the medial side for points of flexion 0^o^ and 30^o^ for the sTKA knee arthroplasty group whereas at the same points of flexion load were greater on the lateral side for the rTKA suggesting a redistribution of load in this range between the two groups of knee procedures. The change in load imbalance was significantly greater in sTKA knees at flexion of 0^o^–60^o^ (s.e. 24) and decreased at a statistically significantly rate for the rTKA knees (− 0.56 s.e. 0.22). This resulted in a knee that would be defined as unbalanced from 0^o^ to 30^o^ with < 15lbs difference in load from medial to lateral compartments. Beyond 60^o^ of flexion the medial to lateral loading equalised to within 15 lbs of pressure for both s-TKA and r-TKS.

**Fig. 7:**
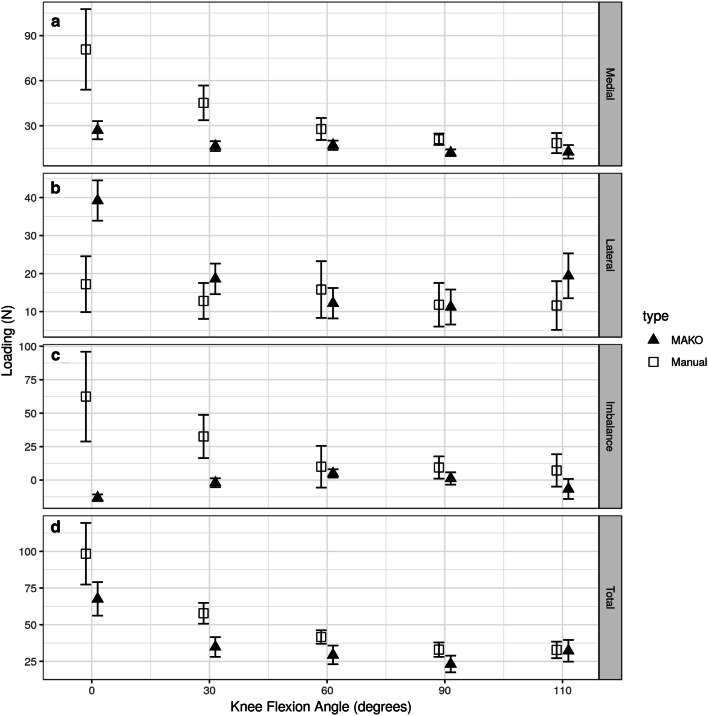
Load across medial and lateral compartments and difference by knee flexion angle for robot-assisted (MAKO) versus conventional (manual) total knee arthroplasty constructs. Black triangles represent MAKO and squares represent manual measurement

### Contact point data

**Fig. 8:**
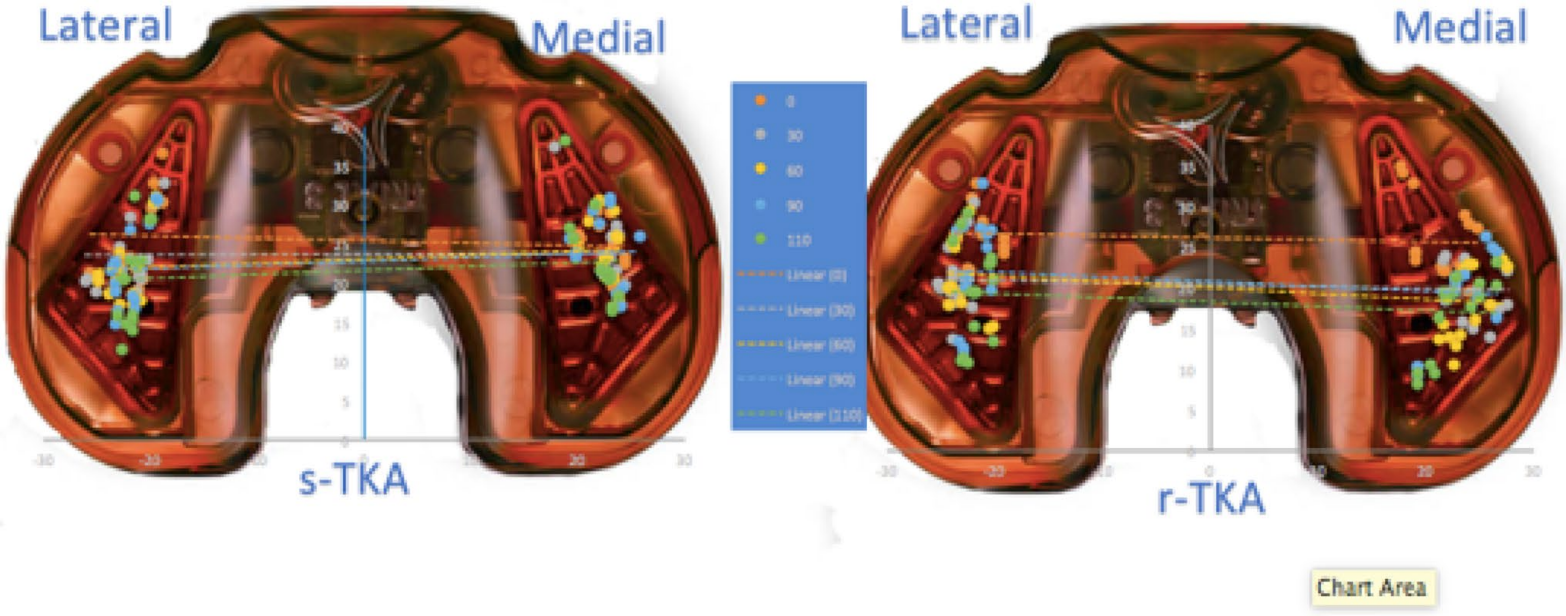
Aerial view of the orthosensor fixed tibial insert with the grid overlay on which the points of contact on both the medial and lateral tibial articular surface were plotted for both rTKA and sTKA components

A grid reference was used to localise the point of contact on the medial and lateral side these points of contact for each position of flexion for the two states of implantation are shown in Fig. 8. For the sTKA it can be seen that the median point of contact on the lateral tibial plateau moved posteriorly incrementally with flexion where the medial point of contact remained relatively constant with flexion of the knee. In contrast, for the rTKA there was a parallel movement with flexion on the medial and lateral plateau posteriorly with no evidence of a pivot effect.

### CT measurements

Raw data for each knee for all measurements are given in Table 1. No intergroup significant differences were found for component alignment or rotation positioning with very similar concordance for the *α*, *β*, *γ* and *δ* angles.

**Table 1:** The final angles for the individual knee arthroplasty CT scan measurements

Specimen	Side	PCL	Akagi	Tibial baseplate	Tibial stem	Alpha	Beta	Gamma	Delta	
NSTC180366	R	− 1	− 5.6	− 5.4	− 5.4	95.4	87.9	2.7	*89*	Robot-assisted
	L	− 7.5	− 5.3	− 8.8	− 7.6	98.8	88.8	− 5	85.1	Conventional
NSTC180393	R	2.1	− 0.3	0	− 0.3	98.6	88.7	− 0.8	92.5	Conventional
	L	12.6	2.7	5.5	5.7	97.4	88.5	− 2.2	87.7	Robot-assisted
NSTCS180298	R	− 4.5	− 8.6	− 4.5	− 5.2	97.2	91.6	7	86.8	Conventional
	L	− 0.6	− 14.4	− 10.1	− 8.2	96.1	91.6	− 0.6	87.4	Robot-assisted
NSTCS180396	R	2.1	− 13.9	4.5	4.7	96	90.7	4.5	87	Robot-assisted
	L	9.6	8.7	7.2	9.8	93.6	91	− 1.1	85	Conventional
NSTCS180406	R	4.1	13.7	2	1.7	101.7	90.8	3.55	87.8	Conventional
	L	− 3.6	− 19	− 2.4	− 3	92.4	90.1	0.4	86.9	Robot-assisted

Femoral Component Rotation (FCR) is the angle subtended by the transepicondylar axis and the posterior condylar line (PCL). The Akagi angle is that between the PCL and the sagittal ruler. The tibial base angle is that angle between a perpendicular to the posterior condylar axis and sagittal ruler. The tibial stem angle is that angle between a perpendicular to the transverse stem axis and sagittal ruler

## Discussion

The key findings of this work were that robotically assisted TKA (rTKA), as opposed to conventional measured resection sTKA, demonstrated consistent equal load distribution between the medial and lateral compartments through a functional arc of motion. Point of contact for the tibiofemoral articulation analysis confirmed the accepted pattern of external tibial rotation with flexion for the sTKA group with parallel movement of the point of the contact posteriorly across the medial and lateral compartments for the rTKA.

Key limitations to the interpretation of this work are that specimens were non-arthritic with no evidence of chondral loss or soft tissue contracture. With advanced arthritic deformity, anatomical landmark identification may not be so straightforward [30, 32]. For the purposes of replicating the in vivo situation muscle loading was reduced to 66.7 N per muscle group to maintain physiological line of pull and replicate the in vivo setting [15, 22]. This is in keeping with previously published works thereby avoiding overloading the sensor. This loading was that below physiological loading but concords with previous work confirming high loads across the medial compartment in cadaveric work particularly near extension [41].

Restoration of correct alignment with restoration of mechanical axis has been shown to reduce radiological loosening and promote implant longevity [20, 23, 26]. This experimental work found no difference using CT-based measures for component placement between conventional or robot-assisted TKA but this was a controlled cadaveric experiment with small numbers and not powered to identify such a difference. Robot-guided bone resection was promoted with the promise of limited surgical exposure, increased accuracy and better alignment and improved performance [24, 38]. Yet, debate continues as to the prognostic value of static mechanical alignment measures for PROMS related clinical performance [25]. Alignment infers load only in the static phase but does not reflect load transfer in flexion [32]. Intra-operative pressure sensors perform real-time full arc load measurement across the two tibiofemoral compartments. Classical teaching argues for equilibration of force in the frontal plane across the two compartments [15, 19, 20]. Up to 20% of mechanically aligned knee arthroplasty cases exhibit load imbalance of greater than 66.7 N load [15, 16]. In this study, with no medial release, the majority of sTKA demonstrated significantly higher medial loads in early flexion. This figure may be even higher when examining torque forces near full extension and may reflect the preservation of the medial soft tissue envelope and in particular the deep component of the medial collateral ligament near the joint margin [41]. Kinematically aligned knees aim to minimize soft tissue release but presume that the soft tissue envelope is stable and will optimize load transfer [18]. This work has confirmed the absence of mediolateral load inequality at time zero for the robotic group of cadaveric knee arthroplasty procedures. Furthermore, one could argue that the parallel movement with flexion limits the torsional effect of tibiofemoral motion as opposed to a medial pivot pattern. This work has extended our knowledge of actual load across the tibiofemoral joint and argues for robotically assisted knee arthroplasty to optimize total load across the joint.

This study found a consistent pattern, under equivalent testing conditions, of progressive axial external rotation of the tibia through flexion for the conventional knee arthroplasty. It is known that such behaviour, whilst predictable in the native knee and desirable in the replaced knee, is inconsistently found [8]. The robot-assisted group in our study demonstrated a standard pattern of posterior sequential translation with rotation through flexion. This is not the same as reverse axial rotation, which has also been found paradoxically within subgroups of knee designs. Reverse axial rotation may expose the knee to patellofemoral instability secondary to lateralization of the tibial tubercle during deep flexion. We did not find any such pattern in any knee in this work and the current study is the first to examine the interrelationship between load transfer and point of contact for robotic total knee arthroplasty. Posterior translation of the tibial point of contact was demonstrated equally with flexion for the medial and lateral compartments in the rTKA subgroup. Previous authors have argued that this pattern is akin to that found in kinematically aligned knees thereby facilitating flexion [5, 42]. The robot-assisted knee arthroplasty exhibited an almost equivalent medial and lateral compartment posterior point of contact movement. Nakamura et al. found in a modelling analysis that the amount of posterior translation at the lateral side in kinematically aligned knee arthroplasty was greater than in the mechanically aligned knee arthroplasty model [31]. However, extrapolation of such time zero behaviour to improved clinical performance remains untested [9, 19]. Furthermore, the CT rotational data suggested a more internally rotated tibial component in the rTKA group, but restoration of mechanical alignment, and such a starting point could help explain the differing modes of femorotibial motion in flexion. Preservation of the soft-tissue envelope whilst restoring alignment and equalising load may offer the potential for improved clinical performance after TKA [44].

## Conclusion

This study has compared the biomechanical behaviour under load of a robot-assisted versus measured resection knee arthroplasty. As such, the robotic group achieved equivalence of load transfer between the two compartments. Laxity was similar for the two implanted states. No significant differences were found for implant positioning determined by CT analyses. Point of motion was parallel and there was no medial pivot in the robotic group.

## References

[1] Abdel MP, Parratte S, Blanc G, Ollivier M, Ponero V, Viehweger E, Argenson JN (2014). No benefit of patient specific instrumentation in TKA on functional and gait outcomes: a randomised clinical trial. Clin Orthop Relat Res.

[2] Akagi M, Yamashita E, Nakagawa T, Asano T, Nakamura T (2001). Relationship between frontal knee alignment and reference axes in the distal femur. Clin Orthop Relat Res.

[3] Baker PN, Van Der Meulen JH, Lewsey J, Gregg PJ (2007). The role of pain and function in determining patient satisfaction after total knee replacement. Data from the National Joint Registry for England and Wales. J Bone Jt Surg [Br].

[4] Bates D, Maechler M, Bolker B, Walker S (2015). Fitting Linear Mixed-Effects Models Using lme4. J Stat Softw.

[5] Belvedere C, Leardini A, Ensini A, Bianchi L, Catani F, Giannini S (2009). Three-dimensional patellar motion at the natural knee during passive flexion/extension. An in vitro study. J Orthop Res.

[6] Calliess T, Bauer K, Stukenborg-Colsman C, Windhagen H, Budde S, Ettinger M (2017). PSI kinematic versus non PSI mechanical alignment in total knee arthroplasty: a prospective, randomized study. Knee Surg Sports Traumatol Arthrosc.

[7] Cheng T, Zhao S, Peng X, Zhang X (2012). Does computer-assisted surgery improve postoperative leg alignment and implant positioning following total knee arthroplasty? A meta-analysis of randomized controlled trials?. Knee Surg Sports Traumatol.

[8] Dennis DA, Komistek RD, Scuderi GR, Zingde S (2007). Factors affecting flexion after total knee arthroplasty. Clin Orthop Relat Res.

[9] Dossett HG, Estrada NA, Swartz GJ, LeFevre GW, Kwasman BG (2014). A randomized controlled trial of kinematically and mechanically aligned total knee replacements: two year clinical results. Bone Jt J.

[10] Elfring R, De la Fuebte M, Radermacher K (2010). Assessment of optical localizer accuracy for computer aided surgery systems. Comput Aided Surg.

[11] Ferket BS, Feldman Z, Zhou J, Oei EH, Bierma-Zeinstra SM, Mazumdar M (2017). Impact of total knee replacement practice: cost effectiveness analysis of data from the osteoarthritis initiative. BMJ.

[12] Ghosh KM, Blain AP, Longstaff L, Rushton S, Amis AA, Deehan DJ (2014). Can we define envelope of laxity during navigated knee arthroplasty?. Knee Surg Sports Traumatol Arthrosc.

[13] Gilmour A, MacLean AD, Rowe PJ, Banger MS, Donnelly I, Jones BG, Blyth MJG (2018). Robotic-arm-assisted vs conventional unicompartmental knee arthroplasty. The 2-year clinical outcomes of a randomized controlled trial. J Arthroplasty.

[14] Grood ES, Suntay WJ (1983). A joint coordinate system for the clinical description of three-dimensional motions: application to the knee. J Biomech Eng.

[15] Gustke KA, Golladay GJ, Roche MW, Elson LC, Anderson CR (2014). A new method for defining balance: promising short-term clinical outcomes of sensor-guided TKA. J Arthroplasty.

[16] Gustke KA, Golladay GJ, Roche MW, Elson LC, Anderson CR (2017). A targeted approach to ligament balancing using kinetic sensors. J Arthroplasty.

[17] Hirschmann MT, Konala P, Amsler F, Iranpour F, Friederich NF, Cobb JP (2011). The position and orientation of total knee replacement components: a comparison of conventional radiographs, transverse 2D-CT slices and 3D-CT reconstruction. J Bone Jt Surg [Br].

[18] Howell SM, Howell SJ, Kt Kuznik, Cohen J, Hull ML (2013). Does a kinematically aligned total knee arthroplasty restore function without failure regardless of alignment category?. Clin Orthop Relat Res.

[19] Howell SM, Hodapp EE, Vernace JV, Hull ML, Meade TD (2013). Are undesirable contact kinematics minimised after kinematically aligned total knee arthroplasty? An intersurgeon analysis of consecutive patients. Knee Surg Sports Traumatol Arthrosc.

[20] Hsu HP, Garg A, Walker PS, Spector M, Ewald FC (1989). Effect of knee component alignment on tibial load distribution with clinical correlation. Clin Orthop Relat Res.

[21] Huang T, Long Y, George D, Wang W (2017). Meta-analysis of gap balancing versus measured resection techniques in total knee arthroplasty. Bone Jt J.

[22] Hunt NC, Ghosh KM, Athwal KK, Longstaff LM, Amis AA, Deehan DJ (2014). Lack of evidence to support present medial release methods in total knee arthroplasty. Knee Surg Sports Traumatol Arthrosc.

[23] Jeffery RS, Morris RW, Denham RA (1991). Coronal alignment after total knee replacement. J Bone Jt Surg [Br].

[24] Jenny JY, Boeri C (2001). Computer assisted implantation of a total knee arthroplasty: a case controlled study in comparison with classical instrumentation. Rev Chir Orthop Repar Appar Mot.

[25] Jones CW, Jerabek SA (2018). Current role of computer navigation in total knee arthroplasty. J Arthroplasty.

[26] Kurtz S, Ong K, Lau E, Mowat F, Halpern M (2007). Projections of primary and revision hip and knee arthroplasty in the United States from 2005 to 2030. J Bone Jt Surg [Am].

[27] Kuznetsova A, Brockhoff PB, Christensen RHB (2017). lmerTest package: tests in linear mixed effects models. J Stat Softw.

[28] Manning WA, Ghosh KM, Blain A, Longstaff L, Rushton SP, Deehan DJ (2018). Internal femoral component rotation adversely influences load transfer in total knee arthroplasty: a cadaveric navigated study using the Verasense device. Knee Surg Sports Traumatol Arthrosc.

[29] Manning W, Blain A, Longstaff L, Deehan DJ (2018). A load measuring device can achieve fine-tuning of mediolateral load at knee arthroplasty but may lead to a more lax knee state. Knee Surg Sports Traumatol Arthrosc.

[30] Moro-oka TA, Shiraishi H, Iwamoto Y, Banks SA (2010). Modified gap-balancing technique in total knee arthroplasty: evaluation of the post-operative coronal laxity. Knee Surg Sports Traumatol Arthrosc.

[31] Nakamura S, Tian Y, Tanaka Y, Kuriyama S, Ito H, Furu M, Matsuda S (2017). The effects of kinematically aligned total knee arthroplasty on stress at the medial tibia: a case study for varus knee. Bone Jt Res.

[32] Park SE, Lee CT (2007). Comparison of robotic assisted and conventional manual implantation of a primary total knee arthroplasty. J Arthroplasty.

[33] Pearle AD, van der List JP, Lee L, Coon TM, Borus TA, Roche MW (2017). Survivorship and patient satisfaction of robotic-assisted medial unicompartmental knee arthroplasty at a minimum two-year follow-up. Knee.

[34] R Core Team (2018) R: a language and environment for statistical computing. R Foundation for Statistical Computing, Vienna. https://www.R-project.org/

[35] Ritter MA, Faris PM, Keating EM, Meding JB (1994). Postoperative alignment of total knee replacement. Its effect on survival. Clin Orthop Relat Res.

[36] Rossi R, Bruzzone M, Bonasia DE, Marmotti A, Castoldi F (2010). Evaluation of tibial rotational alignment in total knee arthroplasty: a cadaver study. Knee Surg Sports Traumatol Arthrosc.

[37] Saffarini M, Nover L, Tandogan R, Becker R, Moser LB, Hirschmann MT, Indelli PF (2019). The original Akagi line is the most reliable: a systematic review of landmarks for rotational alignment of the tibial component in TKA. Knee Surg Sports Traumatol Arthrosc.

[38] Spencer JM, Chauhan SK, Sloan K, Taylor A, Beaver RJ (2007). Computer navigation versus conventional total knee replacement: no difference in functional results at two years. J Bone Jt Surg [Br].

[39] Stoddard JE, Deehan DJ, Bull AM, McCaskie AW, Amis AA (2013). The kinematics and stability of single-radius versus multi-radius femoral components related to midrange instability after TKA. J Orthop Res.

[40] Van der List JP, Chawla H, Joskowicz L, Pearle AD (2016). Current state of computer navigation and robotics in unicompartmental and total knee arthroplasty: a systematic review with meta-analysis. Knee Surg Sports Traumatol Arthrosc.

[41] Verstraete MA, Meere PA, Salvadore G, Victor J, Walker PS (2017). Contact forces in the tibiofemoral joint from soft tissue tensions: implications to soft tissue balancing in total knee arthroplasty. J Biomech.

[42] Watanabe T, Ishizuki M, Muneta T, Banks SA (2013). Knee kinematics in anterior cruciate ligament-substituting arthroplasty with or without the posterior cruciate ligament. J Arthroplasty.

[43] Waterson HB, Clement ND, Eyres KS, Mandalia VI, Toms AD (2016). The early outcome of kinematic versus mechanical alignment in total knee arthroplasty: a prospective randomised control trial. Bone Jt J.

[44] Yang G, Huang W, Xie W, Liu Z, Zheng M, Hu Y, Tian J (2016). Patellar non-eversion in primary TKA reduces the complication rate. Knee Surg Sports Traumatol Arthrosc.

[45] Yin L, Chen K, Guo L, Cheng L, Wang F, Yang L (2015). Identifying the functional flexion-extension axis of the knee: an in-vivo kinematics study. PLoS One 3.

[46] Zambianchi F, Franceschi G, Rivi E, Banchelli F, Marcovigi A, Nardacchione R, Ensini A, Catani F (2019). Does component placement affect short-term clinical outcome in robotic-arm assisted unicompartmental knee arthroplasty?. Bone Jt J.

